# Wound Healing Effect of Gintonin Involves Lysophosphatidic Acid Receptor/Vascular Endothelial Growth Factor Signaling Pathway in Keratinocytes

**DOI:** 10.3390/ijms221810155

**Published:** 2021-09-21

**Authors:** Sun-Hye Choi, Kyung-Jong Won, Rami Lee, Han-Sung Cho, Sung-Hee Hwang, Seung-Yeol Nah

**Affiliations:** 1 Ginsentology Research Laboratory and Department of Physiology, College of Veterinary Medicine, Konkuk University, Seoul 05029, Korea; vettman@naver.com (S.-H.C.); rmlee12@konkuk.ac.kr (R.L.); earth02@konkuk.ac.kr (H.-S.C.); 2 Department of Physiology and Medical Science, School of Medicine, Konkuk University, Seoul 05029, Korea; kjwon@kku.ac.kr; 3 Department of Pharmaceutical Engineering, College of Health Sciences, Sangji University, Wonju 26339, Korea

**Keywords:** gintonin, skin, keratinocyte, proliferation, migration, wound healing

## Abstract

Gintonin, a novel compound of ginseng, is a ligand of the lysophosphatidic acid (LPA) receptor. The in vitro and in vivo skin wound healing effects of gintonin remain unknown. Therefore, the objective of this study was to investigate the effects of gintonin on wound healing-linked responses, especially migration and proliferation, in skin keratinocytes HaCaT. In this study, 2,3-bis-(2-methoxy-4-nitro-5-sulfophenyl)-2H-tetrazolium-5-carboxanilide assay, Boyden chamber migration assay, scratch wound healing assay, and Western blot assay were performed. A tail wound mouse model was used for the in vivo test. Gintonin increased proliferation, migration, and scratch closure in HaCaT cells. It also increased the release of vascular endothelial growth factor (VEGF) in HaCaT cells. However, these increases, induced by gintonin, were markedly blocked by treatment with Ki16425, an LPA inhibitor, PD98059, an ERK inhibitor, 1,2-Bis(2-aminophenoxy)ethane-N,N,N′,N′-tetraacetic acid tetrakis (acetoxymethyl ester), a calcium chelator, and U73122, a PLC inhibitor. The VEGF receptor inhibitor axitinib also attenuated gintonin-enhanced HaCaT cell proliferation. Gintonin increased the phosphorylation of AKT and ERK1/2 in HaCaT cells. In addition, gintonin improved tail wound healing in mice. These results indicate that gintonin may promote wound healing through LPA receptor activation and/or VEGF release-mediated downstream signaling pathways. Thus, gintonin could be a beneficial substance to facilitate skin wound healing.

## 1. Introduction

Ginseng is a traditional herbal medicine that has been used as a tonic for thousands of years. Substantial scientific research has shown that ginseng and its components exert various pharmacological activities, including anti-cancer, anti-diabetes, anti-oxidant, anti-inflammatory, immune system-improving, and central nervous system-improving effects [1,2,3,4,5,6,7,8]. Korean ginseng, a root of *Panax ginseng* C.A. Meyer, contains various biologically active components such as ginsenosides, polysaccharides, and glycolipoproteins [1,9,10]. In a previous study, we isolated a glycolipoprotein called gintonin from ginseng and found that gintonin has a variety of components including ginseng major latex-like protein151 (GLP151), ginseng ribonuclease-like storage protein, phosphatidic acids, and lysophosphatidic acid (LPA) [11]. We also found that it is a ligand of LPA receptor subtypes [11].

LPA receptors are known to be involved in biological effects including mitogenic effects on various cell types, hair follicle development, vascular development, regulation of embryo implantation, and spermatogenesis [12,13]. Gintonin shares many physiological and pharmacological effects with LPA [11,14,15,16]. Gintonin can stimulate intracellular calcium mobilization, proliferation, and migration in various cells [11,14]. It can also protect neuronal cells and improve memory in an Alzheimer’s disease mouse model [15]. Gintonin can also promote hair growth and proliferation of hair follicle cells in mice [16]. Ginseng extracts were also reported to promote skin wound healing [17,18,19]. We have shown that a gintonin-enriched fraction (GEF) of ginseng can enhance hyaluronic acid and collagen release from human skin fibroblasts via LPA receptor activation [20]. In addition, we have shown that gintonin can stimulate vascular endothelial growth factor (VEGF) release from human umbilical vein endothelial cells (HUVEC) [14]. These reports imply that gintonin may affect wound healing-related responses.

Skin wound healing is a very important issue since the skin is an organ and a critical barrier to protect the human body. Thus, many studies have focused on the control of the skin wound healing process. The cutaneous wound healing process has been described in many studies [21,22]. The internal wound healing process is divided into four overlapping phases: hemostasis, inflammation, proliferation, and remodeling [21,22]. Shortly after injury, the hemostasis coagulation process initiates to form a fibrin plug, and platelets aggregate and release various growth factors and cytokines, which play roles in cell recruitment [21,22]. In the inflammation phase, inflammatory cells such as neutrophils, monocytes, macrophages, and dermal cells, such as keratinocytes and fibroblasts are recruited to the wound [21,22]. In the proliferation phase, angiogenesis, extracellular matrix (ECM) deposition, and keratinocyte migration/proliferation are essential events [21,22]. The final remodeling phase of wound healing involves scar formation and revision through collagen remodeling and regression of new capillaries to normal [21,22]. Impaired healing caused by infection, diabetic ulcer, and other chronic wounds may not follow an orderly process of wound healing [21].

Epidermal keratinocytes start migrating to the wound site within a few hours after injury in the inflammation phase [21,22]. Keratinocytes are involved in re-epithelization during the proliferation phase of wound healing and proliferate and differentiate to form a new epithelium [23,24]. Keratinocytes secrete various cytokines to mediate inflammatory responses [25]. Keratinocytes also secrete growth factors, such as transforming growth factor (TGF)-β and VEGF to promote proliferation and migration to initiate angiogenesis [22,25]. Proliferation and/or migration of keratinocytes are stimulated by activating phospholipase C (PLC)/Ca^2+^, extracellular signal-regulated kinase (ERK), phosphoinositide 3-kinases (PI3K)/protein kinase B (AKT), and/or p38 mitogen-activated protein kinases (p38-MAPK) pathways [26,27,28,29,30]. On the other hand, in abnormal conditions, keratinocytes fail to produce appropriate growth factors and disrupt wound healing [31]. Therefore, controlling the biological events of these cell types may be important for promoting wound healing of the skin. Many extracts derived from herbal plants are known to have skin wound healing activity [17,18,19,27,32,33,34]. However, the skin wound healing effect and mechanism of action of gintonin in keratinocytes have not been elucidated yet.

Thus, the objective of this study was to examine the in vitro effects of gintonin on skin wound healing-linked responses using keratinocytes and the in vivo skin wound healing effect of gintonin using a mouse tail wound model. Gintonin treatment enhanced the proliferation and migration of skin keratinocyte HaCaT. The mechanisms of action of gintonin in skin wound healing are also discussed.

## 2. Results

### 2.1. Gintonin-Mediated Enhancement of HaCaT Cell Proliferation and Migration

**Figure 1:**
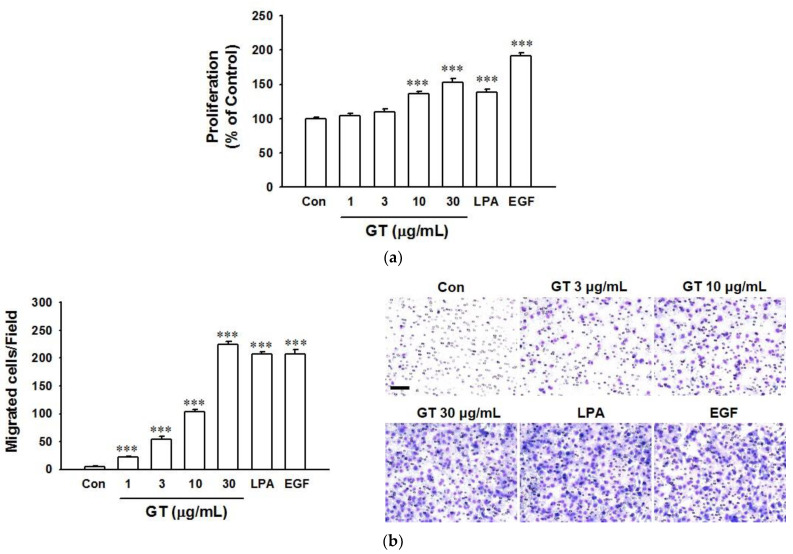
Effects of gintonin on the proliferation and migration of HaCaT cells. (**a**) Cells were incubated in serum-free medium with gintonin (GT; 1–30 μg/mL), lysophosphatidic acid (LPA, 10 μM) as a positive control, or epidermal growth factor (EGF, 4 ng/mL) as another positive control for 24 h. The XTT-based assay was then performed. Response in untreated cells (Con) was considered as 100%. Data represent the means ± S.E.M. (*n* = 6); ***, *p* < 0.001 vs. untreated cell (Con). (**b**) Cells were incubated in serum-free medium for 3 h with GT (1–30 μg/mL), LPA (10 μM) as a positive control, or EGF (4 ng/mL) as another positive control in the modified Boyden chamber. Migration of cells was then analyzed by counting migrated cells as described in the Materials and Methods section. The right panel shows representative images of migrated cells. Scale bar is equivalent to 100 μm. Data represent the means ± S.E.M. (*n* = 16); *** *p* < 0.001 vs. untreated cells (Con). GT, gintonin.

To evaluate the effect of gintonin on skin wound healing, keratinocyte proliferation and migration were tested using a 2,3-bis-(2-methoxy-4-nitro-5-sulfophenyl)-2H-tetrazolium-5-carboxanilide (XTT)-based assay and a modified Boyden chamber (Figure 1a,b), respectively. As shown in Figure 1a, treatment with gintonin at a range of 1–30 μg/mL induced proliferation of HaCaT cells in a dose-dependent manner, peaking at 30 μg/mL. The effect of gintonin at 30 μg/mL in HaCaT cells was similar to that of LPA (10 μM), but was lower than that of epidermal growth factor (EGF, 4 ng/mL) (Figure 1a). Gintonin is known as an LPA receptor ligand and EGF is well known as a skin regeneration factor [11,35,36]. Thus, LPA and EGF were used as positive controls. Gintonin dramatically increased migrated cell numbers with increasing concentrations of gintonin at a range of 1–30 μg/mL. Migrated cell numbers reached the maximum when the gintonin concentration was at 30 μg/mL. The peak effect of gintonin on migrated cell numbers was similar to that of LPA (10 μM) and EGF (4 ng/mL). These results indicate that gintonin might be involved in the initial enhancement of wound healing of skin keratinocytes by inducing proliferation and migration.

### 2.2. In Vitro Wound Healing Effect of Gintonin

**Figure 2:**
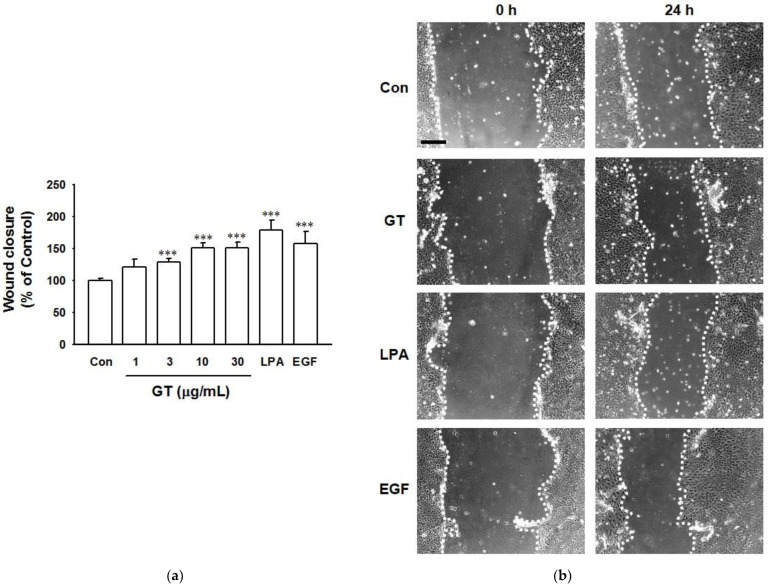
Effects of gintonin on scratch wound healing of HaCaT cells. (**a**) Cells were serum-starved for 24 h. The cell layer was scratched with a micropipette tip to make a wound and then incubated with serum-free medium for 24 h containing gintonin (GT; 1–30 μg/mL), lysophosphatidic acid (LPA, 10 μM), or epidermal growth factor (EGF, 4 ng/mL). Photos were taken at 0 h and 24 h after treatment with GT, LPA, or EGF. (**b**) Representative images of untreated control cells and cells treated gintonin (10 μg/mL). Each cell-free area was outlined with a white dotted line. Scale bar is equivalent to 200 μm. LPA and EGF were used as positive controls. Response of untreated cells (Con) was considered as 100%. Data are presented as the means ± S.E.M. (*n* = 9); *** *p* < 0.001 vs. untreated cells.

The in vitro wound healing effect of gintonin was evaluated by the scratch wound healing assay using HaCaT cells (Figure 2). Gintonin significantly increased the wound closing area at 1–30 μg/mL in a concentration-dependent manner, reaching a plateau at 10 μg/mL. This result indicates that gintonin has a wound-healing effect. The wound-healing effect of gintonin was comparable to that of LPA (10 μM) or EGF (4 ng/mL).

### 2.3. Involvement of LPA1 Receptor Subtypes in Gintonin-Mediated HaCaT Cell Proliferation

**Figure 3:**
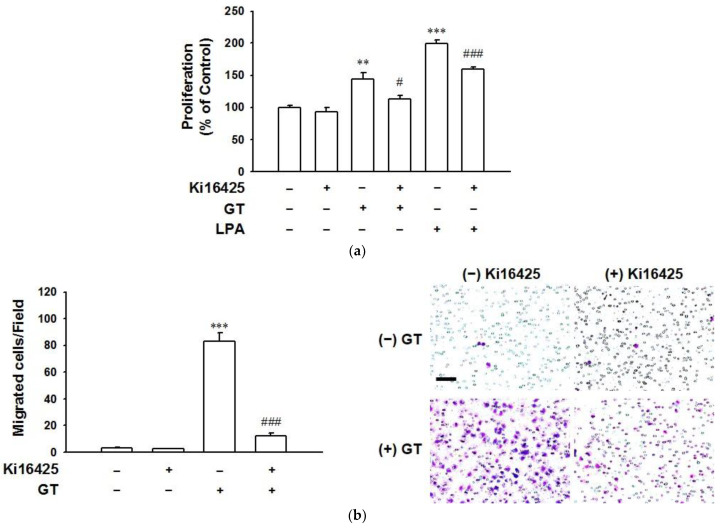
Effect of LPA receptor inhibitor on the proliferation and migration of HaCaT cells. (**a**) Cells were incubated in serum-free medium with gintonin (GT, 10 μg/mL) or lysophosphatidic acid (LPA, 10 μM) in the presence or absence of LPA receptor inhibitor Ki16425 (10 μM) for 24 h. Then, the XTT-based assay was performed. LPA was used as a positive control. Response of untreated cells was considered as 100%. Data are presented as the means ± S.E.M. (*n* = 6); ** *p* < 0.01; *** *p* < 0.001 vs. untreated cells; ^#^ *p* < 0.05; ^###^ *p* < 0.001 vs. GT or LPA alone. (**b**) Cells were incubated with serum-free medium containing GT (10 μg/mL) in the presence or absence of LPA receptor inhibitor Ki16425 (10 μM) for 3 h in the modified Boyden chamber. Cell migration was then analyzed as described in the Materials and Methods section. The right panel shows representative images of migrated cells. Scale bar is equivalent to 100 μm. Data are presented as the means ± S.E.M. (*n* = 16); *** *p* < 0.001 vs. untreated cells; ^###^ *p* < 0.001 vs. GT alone.

To examine whether the effect of gintonin was related to LPA receptor activation, we first examined expression levels of six LPA receptor subtypes in HaCaT cells using immunoblotting. As shown in Figure S1, LPA1/6 receptor subtypes were dominantly expressed in HaCaT cells. We then treated cells with gintonin (10 μg/mL) in the presence or absence of LPA1/3 receptor inhibitor Ki16425. A treatment concentration of 10 μg/mL of gintonin was chosen because the in vitro wound healing effect of gintonin reached a plateau at this concentration. Treatment with Ki16425 (10 μM) significantly attenuated gintonin-induced proliferation and migration of HaCaT cells (Figure 3a,b, respectively). These results indicate that gintonin can induce proliferation and migration via the activation of the LPA1/3 receptor subtype.

### 2.4. Signaling Pathways of Gintonin Effect

**Figure 4:**
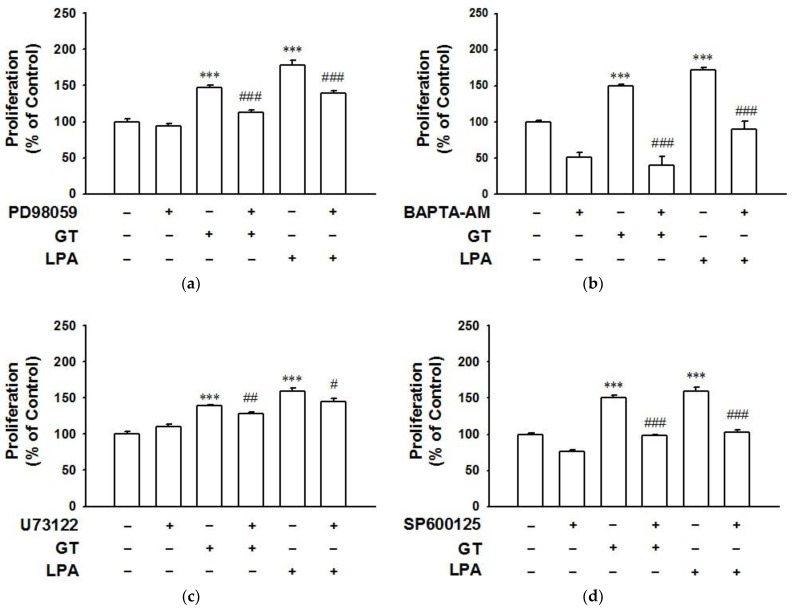
Effects of inhibitors on gintonin-induced proliferation of HaCaT cells. Cells were incubated in serum-free medium containing gintonin (GT, 10 μg/mL) or lysophosphatidic acid (LPA, 10 μM) in the presence or absence of inhibitors for 24 h. Then, the XTT-based assay was performed. Inhibitors used were: ERK inhibitor PD98059 (10 μM) (**a**), calcium chelator BAPTA-AM (10 μM) (**b**), PLC inhibitor U73122 (5 μM) (**c**), and JNK inhibitor SP600125 (20 μM) (**d**). LPA was used as a positive control. Response of untreated cells was considered as 100%. Data are presented as the means ± S.E.M. (*n* = 6); *** *p* < 0.001 vs. untreated cells; ^#^ *p* < 0.05; ^##^ *p* < 0.01; ^###^ *p* < 0.001 vs. GT or LPA alone.

To clarify the downstream signaling pathway of gintonin-mediated activation of LPA receptor, HaCaT cells were treated with gintonin (10 μg/mL) in the presence or absence of inhibitors such as PD98059, an ERK inhibitor, 1,2-Bis(2-aminophenoxy)ethane-N,N,N′,N′-tetraacetic acid tetrakis(acetoxymethyl ester) (BAPTA-AM), an intracellular calcium chelator, U73122, a PLC inhibitor, SP600125, a c-Jun N-terminal kinase (JNK) inhibitor, and LY294002, a PI3K inhibitor. PD98059 (10 μM), BAPTA-AM (10 μM), U73122 (5 μM), and SP600125 (20 μM) significantly blocked gintonin-induced cell proliferation and inhibited LPA-induced proliferation similarly to gintonin (Figure 4a–d). LY294002 (25 μM) also blocked gintonin-induced cell proliferation (Figure S2).

### 2.5. Phosphorylation of Akt and ERK in HaCaT Cells Exposed to Gintonin

**Figure 5:**
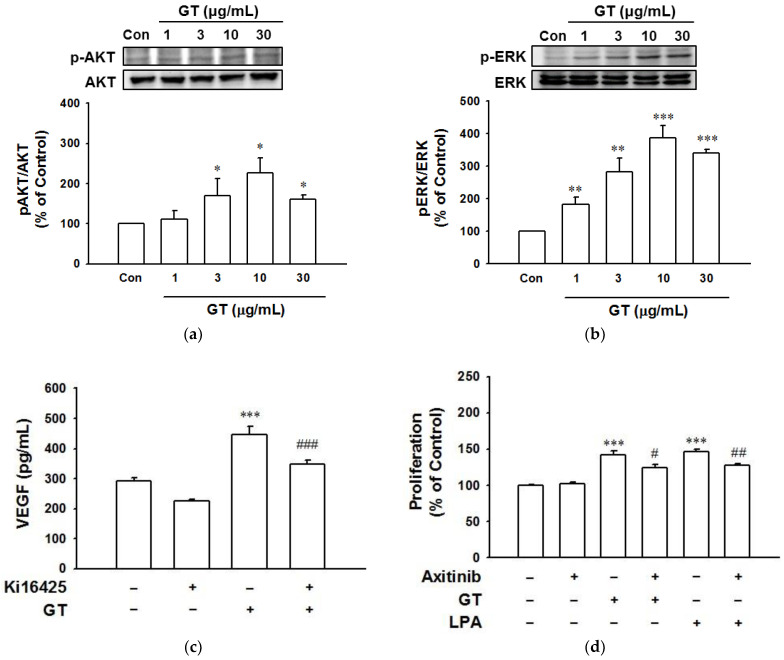
Activation of kinases and changes in receptor inhibitor-induced vascular endothelial growth factor (VEGF) release and proliferation of gintonin-treated HaCaT cells. (**a**,**b**) Cells were incubated in serum-free medium containing gintonin (GT, 1–30 μg/mL) for 10 min and harvested for immunoblotting. extracellular signal-regulated kinase (ERK) 1/2 (**a**) and protein kinase B (AKT) proteins (**b**) were detected using corresponding antibodies. Each protein level in untreated cells (Con) was considered as 100%. Data are presented as the means ± S.E.M. (*n* = 4); * *p* < 0.05; ** *p* < 0.01; *** *p* < 0.001 vs. untreated cells. (**c**) Cells were incubated in serum-free medium containing gintonin (GT, 10 μg/mL) in the presence or absence of LPA receptor inhibitor Ki16425 (10 μM) for 24 h. The VEGF level in the conditioned medium was measured using the ELISA kit as described in the Materials and Methods section. Data are presented as the means ± S.E.M. (*n* = 4); *** *p* < 0.001 vs. untreated cells; ^###^ *p* < 0.001 vs. GT alone. (**d**) Cells were incubated in serum-free medium with gintonin (GT, 10 μg/mL) or lysophosphatidic acid (LPA, 10 μM) in the presence or absence of VEGF receptor inhibitor axitinib (10 μM) for 24 h. Then, the XTT-based proliferation assay was performed. LPA was used as a positive control. Response of untreated cells was considered as 100%. Data are presented as the means ± S.E.M. (*n* = 6); *** *p* < 0.001 vs. untreated cells; ^#^ *p* < 0.05; ^##^ *p* < 0.01 vs. GT or LPA alone. p-ERK, phospho-ERK1/2; p-AKT, phospho-AKT.

To determine the role of phosphorylation of kinases in gintonin-induced proliferation and migration in keratinocytes for wound healing processes, we evaluated the phosphorylation of AKT and ERK1/2 in HaCaT cells exposed to gintonin (1–30 μg/mL) using immunoblotting. Gintonin significantly increased the phosphorylation of AKT and ERK1/2 at 3 to 30 μg/mL, showing the maximum increasing effect at 10 μg/mL (Figure 5a,b). These results indicate that the activation of those proteins by gintonin is involved in the migration and proliferation of keratinocytes associated with wound healing.

### 2.6. Increased VEGF Release from HaCaT Cells Exposed to Gintonin

It is known that gintonin is an LPA receptor ligand and that LPA can increase VEGF release in various cell types [11,16,37]. Moreover, VEGF can be secreted by keratinocytes [22]. We thus examined whether gintonin could stimulate VEGF release from HaCaT cells by an immunoassay. As shown in Figure 5c,d, gintonin (10 μg/mL) significantly induced VEGF release from HaCaT cells. However, the gintonin-induced VEGF release was markedly inhibited by Ki16425 (10 μM) and axitinib (10 μM), showing the involvement of LPA receptor activation in gintonin-enhanced VEGF release and VEGF receptor in keratinocytes.

### 2.7. Gintonin Facilitates In Vivo Wound Repair of Mouse Tail

**Figure 6:**
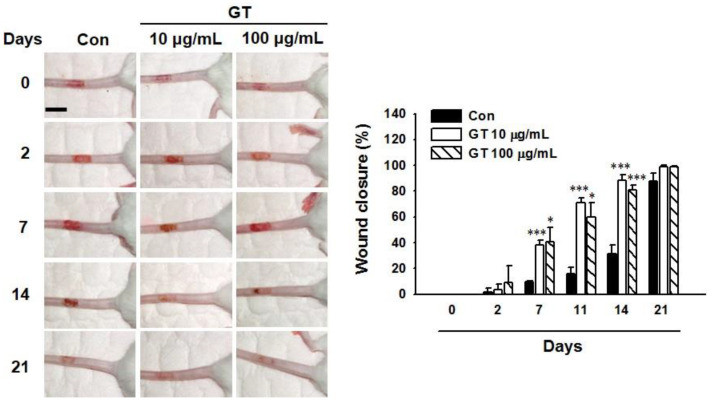
Effects of gintonin on tail wound healing in mice. (**Left panel**) Representative images. Mice were wounded on tails on day 0. Then, the tail wound of mice in each gintonin group was treated with gintonin (GT; 10 μg/mL or 100 μg/mL)-containing cream every 2 or 3 days starting on day 0 for 3 weeks as described in the Materials and Methods section. Tail wounds of mice in the control group (Con) were applied with cream without gintonin on the same day as gintonin treatment. Scale bar is equivalent to 1 cm. (**Right panel**) Graph showing statistical analysis results of wound healing data. Data are presented as the means ± S.E.M. (*n* = 4); * *p* < 0.05, *** *p* < 0.001 vs. control group.

To test the in vivo effect of gintonin, skin wound healing was examined using a mouse tail wound model (Figure 6). Gintonin (10 and 100 μg/mL)-treated groups showed markedly more healing of tail wounds from day 7 to day 14 after being wounded compared to the control group treated with a control cream. Tail wound healing levels were similar for gintonin-treated groups at concentrations of 10 μg/mL and 100 μg/mL. On the other hand, tail wounds in all animal groups recovered almost completely at 21 days after wounding. These results indicate that gintonin treatment may facilitate skin wound healing in mice.

## 3. Discussion

Cutaneous wound healing is an important process to protect the human body. Rapid wound healing and skin regeneration require the use of pharmaceuticals after a skin injury. Many herbal extracts and compounds have been demonstrated to have wound-healing effects on injured skin [17,18,19,27,32,33,34]. Ginseng extract can also promote skin wound healing [17,18,19]. However, its detailed active components and mechanism of action have not yet been clearly explained.

Previously, we have reported that gintonin derived from ginseng is an LPA receptor ligand and that gintonin or GEF can induce intracellular calcium transient, cell proliferation, and migration of several cell types, such as HUVEC, human hair follicle cells, and dermal fibroblasts [11,14,16]. However, it is not known whether gintonin affects keratinocytes for actual wound healing. Proliferation, migration, and scratch wound healing tests are usually used to find skin wound healing effects of a drug [27,29,30,34]. In this study, we found that gintonin increased the proliferation and migration of human keratinocytes HaCaT. Moreover, gintonin-induced closure of in vitro wounds using HaCaT cells showed the ability of gintonin to promote the proliferation and migration of keratinocytes, since the proliferation and migration of keratinocytes are key processes associated with skin wound healing events [30]. Interestingly, in the present study, we found that topical application of a gintonin-containing cream to tail wounds of mice also ameliorated tail wounds more effectively than a control cream without gintonin. These findings imply that gintonin may exert positive effects on skin wound healing.

**Figure 7:**
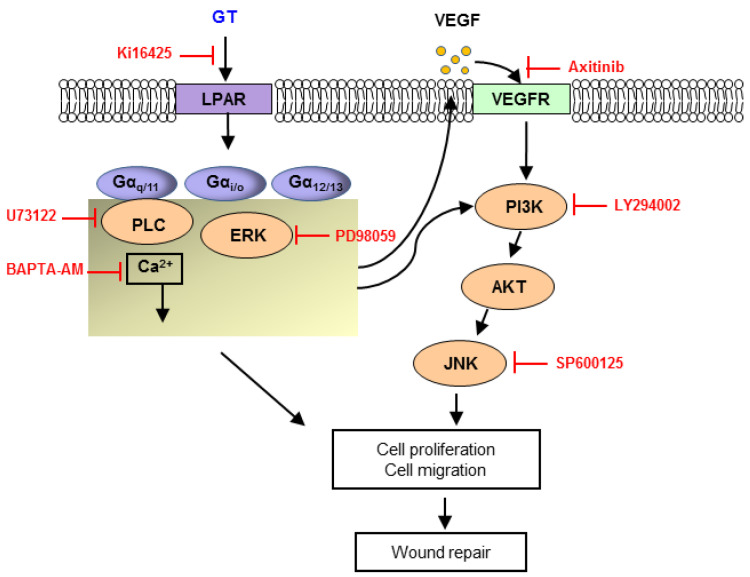
Prospective signaling pathways for gintonin-induced wound repair of keratinocytes. Lysophosphatidic acid (LPA) receptor activation by gintonin (GT) may activate protein kinase C (PKC)/extracellular signal-regulated kinase (ERK), phospholipase C (PLC)/intracellular Ca^2+^ mobilization through G-proteins, leading to keratinocyte proliferation and migration as well as vascular endothelial growth factor (VEGF) release. VEGF may also activate VEGF receptor/phosphoinositide 3-kinases (PI3K)/protein kinase B (AKT)/c-Jun N-terminal kinase (JNK), leading to keratinocyte proliferation and migration. Ultimately, these signaling pathways may promote skin wound repair. LPAR, lysophosphatidic acid receptor; VEGFR, vascular endothelial growth factor receptor.

Here, several experiments were conducted to investigate signaling mechanisms involved in the wound healing effect of gintonin. We found that skin wound-healing signaling inhibitors influenced gintonin-enhanced proliferation and migration, implying that downstream signaling pathways might be involved in the action of gintonin for wound healing (Figure 7). Our results showed that LPA1/3 receptor inhibitor Ki16425 markedly decreased gintonin-induced proliferation and migration. LPA receptors are present in keratinocytes [38,39]. Interestingly, our results confirmed that LPA1-6 receptor subtypes were expressed in HaCaT cells by immunoblotting (Figure S1), although the roles of LPA receptor subtypes in keratinocytes were not clearly known. It has been reported that LPA is released to a wound site from the serum and platelets to induce wound healing-related responses [38,40]. LPA can promote the migration and proliferation of keratinocytes and enhance wound healing in mice and rats [38,41,42]. Taken together, these findings suggest that gintonin can induce the proliferation and migration of keratinocytes through LPA receptor activation.

Moreover, in this study, gintonin increased the phosphorylation of AKT and ERK in keratinocytes. AKT and ERK phosphorylation are well-known cellular events related to cell proliferation. Additionally, ERK inhibitor PD98059, calcium chelator BAPTA-AM, PLC inhibitor U73122, and JNK inhibitor SP600125 all significantly inhibited both gintonin-induced proliferation and LPA-induced proliferation of HaCaT cells. LPA coupled with G proteins (Gαq/11, Gα11/12, Gαi/o, and Gαs) shows differential biological activities, including cell migration, cytoskeleton change, and cell proliferation [12,13]. Major downstream signaling pathways of LPA receptor activation include PLC/Ca^2+^, PLC/protein kinase C (PKC), Ras homolog family member A (Rho A)/ROCK (Rho-associated protein kinase), Ras/mitogen-activated protein kinase kinase (MEK)/ERK, and PI3K/AKT pathways [12,13]. Therefore, gintonin might share AKT/MAPKs and PLC/Ca^2+^ pathways with LPA in keratinocytes for wound healing.

In the wound healing process, keratinocytes can proliferate and migrate to the wound bed and release various cytokines and growth factors including VEGF [22]. LPA increased VEGF release in various cell types [11,37]. Moreover, in a pilot experiment, we found that gintonin increased VEGF release in a concentration-dependent manner in HaCaT cells (data not shown). In the present study, we thus tested the effect of gintonin (an LPA receptor ligand) on VEGF release in HaCaT cells and found that gintonin stimulated VEGF release from HaCaT cells. Moreover, this VEGF release was inhibited by LPA1/3 receptor inhibitor Ki16425, suggesting that the LPA receptor might be involved in gintonin-stimulated VEGF release (Figure 5c). In addition, VEGF receptor inhibitor axitinib partially attenuated the proliferation of keratinocytes (Figure 5d). Taken together, these results suggest that VEGF released from keratinocytes could act as an autocrine bioactive molecule via VEGF receptor activation and partially contribute to the proliferation of HaCaT cells (Figure 7). Moreover, in our previous study, gintonin also enhanced proliferation and collagen release in skin fibroblasts, which are involved in the proliferation and remodeling phases. Gintonin attenuated the expression of proinflammatory cytokines that were released as a result of inflammation in macrophages, indicating its anti-inflammatory effect [43]. The wound healing process includes overlapping complex phases such as hemostasis, inflammation, proliferation, and remodeling [21,22]. These reports, and our results, imply that gintonin may participate in wound healing-related responses at different stages of the wound healing process. Therefore, gintonin may have beneficial effects on healing skin wounds.

On the other hand, EGF has been recognized as an excellent wound healing agent due to its therapeutic functions in stimulating skin cell growth, proliferation, and differentiation [35,36]. In the present study, we found that the wound healing effect of gintonin was comparable to that of EGF (Figure 2). Since signaling pathways of gintonin and EGF for wound healing processes are different from each other, it will be interesting to examine their additive or synergistic effects on in vivo wound healing in the future using a combination of gintonin and EGF.

In conclusion, the present study demonstrated that gintonin enhanced cell proliferation, migration, VEGF release, and wound closure in in vitro assays. Gintonin also promoted in vivo wound healing on mice tail wounds. Gintonin-enhanced cell proliferation, migration, and VEGF release were markedly blocked by treatment with an LPA receptor inhibitor. An ERK inhibitor, a calcium chelator, a PLC inhibitor, and a VEGF receptor inhibitor also attenuated gintonin-enhanced cell proliferation. Gintonin increased the phosphorylation of AKT and ERK1/2. These findings indicate that gintonin may promote wound healing by activating LPA receptor-VEGF release-mediated downstream signaling pathways (Figure 7). Therefore, gintonin, as a bioactive natural component, could be a promising material to be developed for skin wound healing in the future.

## 4. Materials and Methods

### 4.1. Materials

Gintonin was prepared from *Panax ginseng* as previously described [44]. 1-Oleoyl-2-hydroxy-sn-glycero-3-phosphate (LPA C18:1) was purchased from Avanti Polar Lipids (Alabaster, AL, USA). Anti-EDG2 (LPA1) antibody, anti-LPA2 antibody, anti-EDG7 (LPA3) antibody, anti-LPA4 antibody, and anti-β–actin horse radish peroxidase-conjugated antibodies were purchased from Abcam (Cambridge, MA, USA). Anti-LPA5 antibody was purchased from Biorbyt (San Francisco, CA, USA). Anti-P2RY5 (LPA6) antibody was purchased from OriGene Technologies, Inc. (Rockville, MD, USA). Anti-ERK1/2, anti-phospho-ERK1/2, anti-AKT, and anti-phospho-AKT antibodies were purchased from Cell Signaling Technology, Inc. (Danvers, MA, USA). Goat anti-rabbit IgG antibody was purchased from GeneTex (Irvine, CA, USA). An enzyme-linked immunosorbent assay (ELISA) kit for VEGF was purchased from R&D systems (Minneapolis, MN, USA). Ki16425 was purchased from Cayman Chemical (Ann Arbor, MI, USA). Dulbecco’s Modified Eagle’s Medium (DMEM) (low glucose) was purchased from Welgene Inc. (Gyeongsan-si, Gyeongsangbuk-do, Korea). All other materials for cell culture were purchased from Thermo Fisher Scientific Korea (Gangnam-gu, Seoul, Korea). EGF and all other reagents used were purchased from Sigma-Aldrich (St. Louis, MO, USA).

### 4.2. Cell Culture

HaCaT human skin keratinocytes were kindly provided by Prof. HM Lee (Hoseo University, Asan-si, Chungcheongbuk-do, Korea). HaCaT cells were cultured in DMEM supplemented with 10% (*v*/*v*) fetal bovine serum (FBS), 100 units/mL penicillin, and 100 μg/mL streptomycin.

### 4.3. Cell Proliferation Assay

Cell proliferation was measured with the XTT-based assay as previously described [14]. Briefly, cells (5 × 10^3^ cells/well) were seeded into 96-well plates, incubated for 48 h, and were serum-starved for 4 h. These cells were then treated with different concentrations of gintonin, LPA, or EGF for 24 h. In some experiments, inhibitors were used for pretreatment before treatment with gintonin or LPA. The culture medium of cells was replaced with fresh serum-free medium without phenol red. These cells were then treated with a mixture of XTT and phenazine methosulfate (1 mg/mL and 0.03 mg/mL, respectively). The absorbance at 450 nm of orange formazan indicating cell viability relative to the activity of the mitochondrial enzyme was detected with the Spectra Max 190 plate reader (Molecular Devices, Sunnyvale, CA, USA).

### 4.4. Migration Assay

The migration of HaCaT cells across a collagen-coated polycarbonate membrane (8 μm pore size) was measured using modified Boyden chambers (Neuro Probe, Gaithersburg, MD, USA) as previously described [11,14]. Gintonin, LPA, or EGF in serum-free DMEM was added to lower chambers. Cells (5 × 10^4^ cells/well) were loaded into upper chambers and incubated at 37 °C for 3 h. In some experiments, cells were pretreated with or without inhibitors before being loaded into the chamber. Migrated cells were fixed, stained with Diff Quik (Sysmex, Kobe, Japan), and photographed and counted using a dark field microscope (Eclipse 80i; Nikon, Tokyo, Japan) at a magnification of ×200.

### 4.5. Scratch Wound Healing Assay

In vitro wound-healing assay was performed as previously described [14]. Briefly, HaCaT cells (2.5 × 10^5^ cells/well) were seeded into 24-well plates pre-coated with 0.1 mg/mL of collagen type I from rat tails (BD Bioscience, San Jose, CA, USA) and incubated at 37 °C for 24 h. These cells were incubated in serum-free medium at 37 °C for 4 h. Cell layers were scratch-wounded using a 200 μL pipette tip. After washing with serum-free medium, the cells were incubated with different concentrations of gintonin, LPA, or EGF at 37 °C for 24 h. Images were photographed using an inverted fluorescence microscope (AxioVert200; Carl Zeiss, Oberkochen, Germany) at a magnification of ×100 and analyzed using an image analyzing software (OptiView, Jacksonville, FL, USA). Wound closure levels were quantified as follows: The ratio of wound closure in each group was calculated as the recovered area at 24 h divided by the initial wound area (at 0 h). Then, the value obtained in each group was expressed as a percentage of wound closure of untreated control cells.

### 4.6. VEGF ELISA

The VEGF level was detected as previously reported [14] with some modifications. Briefly, HaCaT cells were incubated with serum-free DMEM at 37 °C for 6 h and with DMEM in the presence or absence of gintonin at different concentrations for 24 h. VEGF content in the supernatant of cultured medium (conditioned medium) was measured using an ELISA kit (R&D Systems, Minneapolis, MN, USA) according to the manufacturer’s instructions.

### 4.7. Immunoblotting

Phosphorylation levels of ERK and AKT and LPA subtypes in HaCaT cell lysates were examined. Cells were lysed using a modified radioimmunoprecipitation assay (RIPA) buffer containing protease inhibitors and phosphatase inhibitors. Each protein was detected after 10% sodium dodecyl sulfate polyacrylamide gel electrophoresis (SDS-PAGE) and immunoblotting using rabbit anti-phospho-ERK polyclonal antibody (1:1000), rabbit anti-phospho-AKT antibody (1:1000), and goat anti-rabbit IgG antibody conjugated to HRP. The membrane was stripped and re-probed with rabbit anti-ERK polyclonal antibody (1:1000) and rabbit anti-AKT antibody (1:1000). LPA receptor subtypes LPA1–6 were also detected using LPA1–6 antibodies, as previously described [26]. For detection of loading controls, mouse anti-β-actin monoclonal antibody conjugated to HRP (1:30,000) was also used. Images were visualized using Clarity Western ECL Substrate Bio-Rad (Hercules, CA, USA) with an iBright CL1000 imaging system (Thermo Fischer Scientific, Waltham, MA, USA).

### 4.8. In Vivo Wound Healing

The in vivo wound healing assay was performed using a mouse tail wound model as previously described [45] with some modifications. Briefly, 7-week-old male ICR mice were used. Animal experiments were performed in accordance with the Guide for the Care and Use of Laboratory Animals of Konkuk University (KU21051-1). A rectangular wound (approximately 5 × 10 mm) was made on the dorsal side of each mouse tail using a scalpel and Cautery Kit (Bovie Medical Corporation, Clearwater, FL, USA). The size and shape of each wound were standardized using a template and other conditions were identical. Mice were intramuscularly injected with xylazine (2 mg/kg) before wounding. Gintonin (10 μg/mL or 100 μg/mL)-containing cream was applied on the wound lesion every 2 days starting at day 0 for 21 days after wounding. Cream without gintonin was also used for the control group of mice. Wounds were photographed and wound areas were measured using an image analyzing software (OptiView, Jacksonville, FL, USA).

### 4.9. Statistical Analysis

Data are expressed as the means ± standard deviation. Statistical comparisons of controls and treated experimental groups were performed using Student’s *t*-test or one-way analysis of variance (ANOVA) using GraphPad Prism, version 5.0 (Graph Pad Software, San Diego, CA, USA). *p*-values less than 0.05 were considered statistically significant.

## Supplementary Materials

The following are available online at https://www.mdpi.com/article/10.3390/ijms221810155/s1.
