# Supplementary material for: Wound Healing Effect of Gintonin Involves Lysophosphatidic Acid Receptor/Vascular Endothelial Growth Factor Signaling Pathway in Keratinocytes
Source: Int J Mol Sci. 2021 Sep 21;22(18):10155. doi: 10.3390/ijms221810155 (PMC8467330; doi:10.3390/ijms221810155)
Supplement: Supplementary file 1 [file ijms-22-10155-s001.zip › Choi et al_IJMS_1371978 Supplementary data.pdf]

Supplementary data

Wound healing effect of gintonin involves lysophosphatidic acid receptor/vascular endothelial growth factor signaling pathway in keratinocytes

Sun-Hye Choi[1, †], Kyung-Jong Won[2, †], Rami Lee[1], Han-Sung Cho[1], Sung-Hee Hwang[3,*] and Seung-Yeol Nah[1,*]

[1] Ginsentology Research Laboratory and Department of Physiology, College of Veterinary Medicine, Konkuk University, Seoul 05029, Republic of Korea; vettman@naver.com (S.-H.C.), rmlee12@konkuk.ac.kr (R.L.), earth02@konkuk.ac.kr (H.-S.C), syn-ah@konkuk.ac.kr (S.-Y.N)

[2] Department of Physiology and Medical Science, School of Medicine, Konkuk University, Seoul 05029, Republic of Korea; kjwon@kku.ac.kr (K.-J.W.)

[3] Department of Pharmaceutical Engineering, College of Health Sciences, Sangji University, Wonju, 26339, Republic of Korea; sunghhwang@sangji.ac.kr (S.-H.H)

* Correspondence: synah@konkuk.ac.kr (S.-Y.N), sunghhwang@sangji.ac.kr (S.-H.H); Tel.: +82-2-450-4154 (S.-Y.N), +82-33-738-7922 (S.-H.H)

† These authors contributed equally to this work.

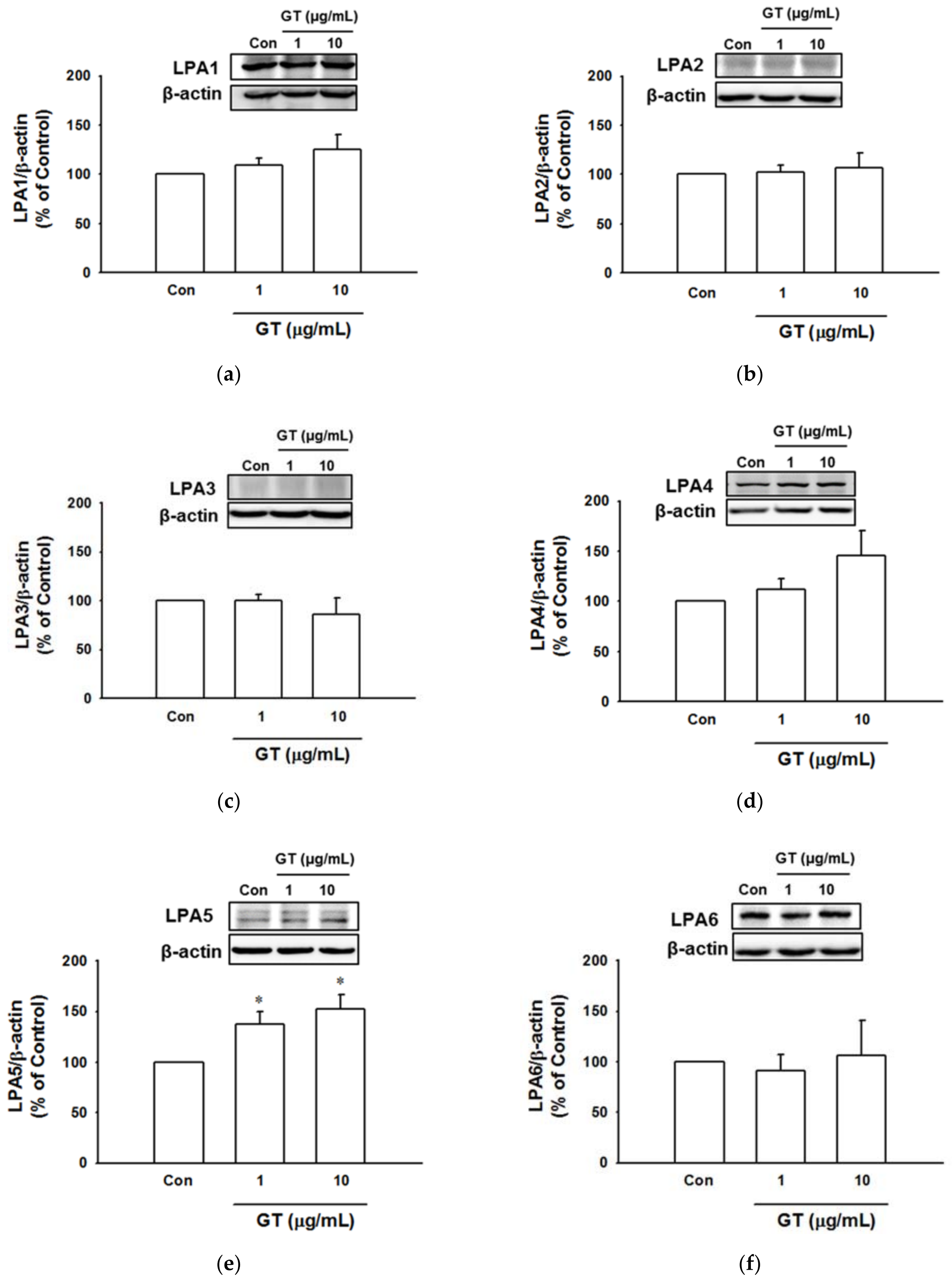

Figure S1. Expression of lysophosphatidic acid (LPA) receptor subtypes in HaCaT cells. Cells were incubated in serum-free medium with gintonin (GT, 1 or 10 μg/mL) for 6 h. Then, the cell lysates were analyzed by immunoblotting, using LPA receptor subtypes LPA1-6 antibodies as described in the Materials and Methods section of the main text. Response in untreated cell (Con) was considered as 100%. Data represent means ± S.E.M. ($n = 6$); $^{*}p < 0.05$, vs. untreated cells.

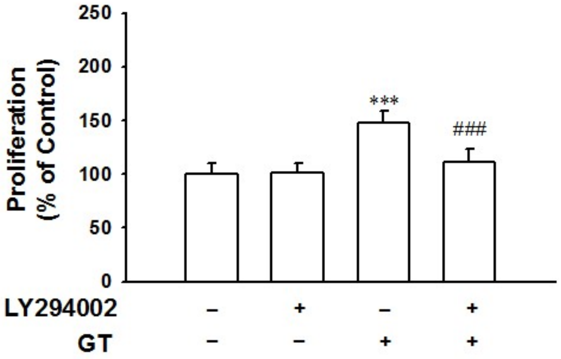

Figure S2. Effect of PI3K inhibitor LY294002 on gintonin-induced proliferation of HaCaT cells. Cells were incubated in serum-free medium with gintonin (GT, 10 μg/mL) in the presence or absence of inhibitor for 24 h. Then, XTT-based assay was performed. LY294002 (25 μM). Response in untreated cell was considered as 100%. Data represent means ± S.E.M. (n = 6); *** $p < 0.001$, vs. untreated cells; ### $p < 0.001$, vs. GT alone.
